# Reports and Announcements Received

**Published:** 1856-09

**Authors:** 


### REPORTS AND ANNOUNCEMENTS RECEIVED.

We find upon our table the "Fifth Annual announcement of Lectures of the Miami Medical College, of Cincinnati, for the Session of 1856–7." They say that "the success of the College has been all its warmest friends could have anticipated. The number of students has been constantly increasing, while the aggregate number in the majority of Schools has diminished."

They report 63 Students and 18 Graduates for the last Session—the next Session commences on the 15th of October, and continues until the 1st of March.

Also the "Report and Announcement of the Medical Department of the University of Pennsylvania, for the Session of 1856–7." The number of Students in attendance during the past Session was 372, and the number of Graduates 142. The 91st Session will commence on Monday, the 13th of October, and be continued until the middle of the following March.

Also the "Circular of the Faculty of Oglethorpe Medical College, at Savannah, Ga., with a Catalogue of the Students and Graduates, and Announcement of Lectures, for the Session of 1856–7."

There seems to have been a re-organization of the Faculty, since the last Session, and in their announcement it is stated that "Its Faculty and friends are determined to make it second to no Medical College in the Country, in the abundant facilities which it shall afford, to impart a thorough Medical education to those who may patronize it. So may it be, and, thus deserving, receive the fullest success—while we desire to see all prosper, we confess to a special interest in the success of Southern Colleges, and with the growing feeling upon the part of the South, that our young men ought to be educated at home, if the proper facilities are offered here, they will assuredly be appreciated sooner or later. Students 38, Graduates 8. Lectures commence 1st Monday in November, and close 1st of March.

We have also the "Annual Circular of the Trustees, Faculty and Students of the Medical College, of the State of South Carolina, for 1855–6," with which the eminent Dickson is connected. The institution seems to be in a very flourishing condition, the Class in attendance upon the last Course of Lectures amounting to 220, and the number of Graduates 86. The next Session commences on the first Monday in November, and terminates on the first Saturday in March following.

